# A TtAgo-mediated LAMP System for highly sensitive and specific detection of rotavirus in pediatric diarrheal samples

**DOI:** 10.3389/fcimb.2025.1682786

**Published:** 2025-10-13

**Authors:** Ligong Wang, Linlin Fan, Dan Wang, Qian Ma, Jian Hu, Xiaoqin Wang

**Affiliations:** Department of Clinical Laboratory, The First Affiliated Hospital of Xi’an Jiaotong University, Xi’an, Shaanxi, China

**Keywords:** LAMP (loop mediated isothermal amplification), AGO (Argonaute), rotavirus (RV), pediatric, diarrheal

## Abstract

Rotavirus remains a leading cause of severe pediatric gastroenteritis worldwide, particularly in regions with limited healthcare resources, timely diagnosis is essential for effective patient management and outbreak containment. Loop-mediated isothermal amplification (LAMP) offers rapid nucleic acid amplification under constant temperature, yet classic LAMP assays are prone to false positives from nonspecific amplification products. Here, we present a reverse transcription LAMP (RT-LAMP) assay integrated with *Thermus thermophilus* Argonaute (TtAgo)–mediated probe cleavage for highly sensitive and specific detection of rotavirus. The assay targets a conserved region of the NSP5 gene, coupling rapid isothermal amplification with programmable guide DNA–directed cleavage and fluorescence signal generation. Under optimized conditions, the system reliably detected as few as 10 copies/μL within 60 minutes, without cross-reactivity to other common viral or bacterial pathogens. In clinical testing of 60 pediatric stool samples (40 rotavirus-positive, 20 negative diagnosed by RT-qPCR), the TtAgo-mediated LAMP assay achieved 100% sensitivity and specificity, outperforming classic LAMP and antigen-based methods. Receiver operating characteristic analysis yielded an area under the curve of 1.00, compared with 0.80 for classic LAMP. These findings demonstrate that integrating TtAgo into the LAMP workflow markedly enhances diagnostic accuracy and reliability, providing a practical and laboratory-ready molecular tool for rotavirus detection.

## Introduction

Rotavirus is a major etiological agent of acute gastroenteritis in children under five years old, causing considerable global morbidity, hospitalization, and mortality (Dhital et al., 2017; Bulut et al., 2018). The disease burden is markedly higher in low- and middle-income countries, where access to advanced healthcare infrastructure is often limited. Although widespread vaccination programs have significantly reduced overall incidence, rotavirus remains a persistent public health concern. Factors such as incomplete immunization coverage, breakthrough infections, and the continual emergence of genetically diverse strains contribute to its sustained prevalence (Sindhu et al., 2017; Cohen et al., 2022). Prompt and accurate diagnosis is therefore essential for guiding clinical interventions, implementing timely infection control, and preventing community-wide outbreaks.

Conventional diagnostic methods for rotavirus include lateral flow immunoassays and reverse transcription polymerase chain reaction (RT-PCR). RT-PCR is widely recognized as the gold standard owing to its high sensitivity and specificity (Zeng et al., 2008). However, its dependence on sophisticated instrumentation, skilled operators, and centralized laboratories limits accessibility in many clinical and field settings (Soroka et al., 2021). Rapid antigen tests offer ease of use but are often limited by suboptimal sensitivity, particularly in cases with low viral loads (Ushijima et al., 2024). These limitations underscore the need for diagnostic platforms that combine high analytical performance with operational simplicity and rapid turnaround.

Loop-mediated isothermal amplification (LAMP) has emerged as a promising nucleic acid amplification technique, enabling rapid target amplification at a constant temperature, typically within one hour, without the need for thermal cycling (Notomi et al., 2000). Its tolerance to crude clinical samples and minimal equipment requirements have made it attractive for pathogen detection (Lee et al., 2024; Xiao et al., 2024). Nevertheless, a recognized shortcoming of classic LAMP assays is their susceptibility to false-positive results, primarily due to nonspecific amplification, target independent amplification and primer dimer formation (Ye et al., 2018a; Lin et al., 2019; Kim et al., 2023). To address this issue, LAMP has been coupled with CRISPR–Cas–based detection, leveraging sequence-specific collateral cleavage to enhance specificity (Atçeken et al., 2022). While effective, CRISPR-based systems generally require a protospacer adjacent motif (PAM) and rely on guide RNA, which imposes inherent constraints on target site selection and poses challenges for the stability and preservation of guide RNAs during storage and transport (Collias and Beisel, 2021).

Argonaute (Ago) proteins offer an alternative approach to enhancing LAMP specificity. Unlike CRISPR nucleases, Ago proteins such as *Thermus thermophilus* Argonaute (TtAgo) are PAM-independent and utilize short 5′-phosphorylated single-stranded DNA guides to cleave complementary nucleic acid targets with high precision (Lin et al., 2022; Wang et al., 2023; Zhang et al., 2024). This flexibility facilitates direct integration with DNA amplification products without transcription or rigorous sequence requirements. When paired with a fluorescence-quenched molecular beacon, TtAgo-mediated cleavage generates a strong, target-dependent fluorescence signal, effectively minimizing false-positive readouts associated with LAMP alone.

In this study, we present a reverse transcription LAMP (RT-LAMP) assay coupled with TtAgo-mediated molecular beacon cleavage for sensitive and specific detection of rotavirus. Targeting a conserved region within the nonstructural protein 5 (NSP5) gene ensure broad genotype coverage. By combining rapid RNA amplification with programmable nucleic acid cleavage and precise fluorescence signal generation, the assay achieves high analytical specificity while maintaining the inherent speed of isothermal amplification. We evaluated its sensitivity, specificity against a panel of common enteric pathogens, and clinical performance using stool samples from pediatric patients with acute diarrhea, aiming to provide a reliable, accurate, and laboratory-ready diagnostic tool for rotavirus detection.

## Materials and methods

### Target selection and synthetic template preparation

A highly conserved region within the rotavirus nonstructural protein 5 (NSP5) gene was selected as the assay target. To ensure specificity, a 661-nt conserved sequence was obtained through multiple sequence alignment of representative rotavirus A strains, followed by BLAST analysis against the NCBI database to exclude homology with non-target organisms. The corresponding DNA fragment (Shanghai Sangon, China) was synthesized and transcribed *in vitro* into RNA using the MEGAscript™ T7 kit (Invitrogen), providing reference material for assay development and analytical sensitivity assessment.

### Design of RT-LAMP primers

LAMP primers targeting the NSP5 gene were designed using PrimerExplorer V5 software, following standard LAMP design rules. The sequences were as follows: F3: 5′-CATAAGAAGGAGAAATCCAAGAA-3′; B3: 5′-CCTAGTGTGCTCTCAGGTTA-3′; FIP: 5′-CGTCATCACTATCTGAATCATCCAAATAAAAGTAGGAAACACTACCCG -3′; BIP: 5′-AAGTATTTCGCACTAAGAATGAGGACATTACAAATCTTCGATCAATTGC-3′.

Primers were HPLC-purified (Shanghai Sangon) and dissolved in nuclease-free water to working concentrations. Multiple sets were screened to maximize amplification efficiency while minimizing primer–dimer formation.

### TtAgo-mediated LAMP reaction system

All reactions were performed in a closed tube to avoid contamination. A 25 μL reaction mixture was prepared containing 1× Thermalpol amplification buffer (New England Biolabs), 8 U Bst DNA polymerase large fragment, 10 U AMV reverse transcriptase (Takara), 1.4 mM dNTPs, 6 mM MgSO^4^, 0.8 M betaine, 0.2 μM each of F3 and B3, 1.6 μM each of FIP and BIP, and 2 μL of template RNA.

For the TtAgo cleavage reaction, 100 nM of purified TtAgo (New England Biolabs) protein was pre-incubated with two 5′-phosphorylated guide DNAs (gDNA1: 5′- TTCTTACATTTACCGT-3′ and gDNA2: 5′- TTTTTCTTATATTTAC-3′), each at 100 nM, in cleavage buffer containing 10 mM Tris-HCl (pH 8.0), 10 mM NaCl, and 0.5 mM MnCl_2_. The RT-LAMP amplicons served as primary targets, generating an 18-nt cleavage product (CP: 5′-AATGTAAGAATTGTAAAT-3′). This CP served as a secondary guide (gDNA3) to direct cleavage of a FAM–BHQ1 molecular beacon probe (5′-FAM-CGCGGCATTTACAATTCTTACATTGCCGCG-BHQ1-3′, 100 nM). Cleavage between bases 10 and 11 separated the fluorophore from the quencher, producing a measurable fluorescence signal.

### Thermal reaction conditions

The complete reaction was carried out in a real-time PCR instrument using the following thermal protocol: 63°C for 45 minutes for RT-LAMP amplification, followed by 80°C for 30 minutes for TtAgo-mediated cleavage, and a final hold at 4°C. Real-time fluorescence (FAM channel) was monitored using a PCR instrument. The positivity threshold was defined as the mean fluorescence of negative controls plus three standard deviations; curves crossing this threshold within the assay window were scored as positive.

### Analytical sensitivity and specificity evaluation

For sensitivity, serial dilutions (10^5–^10^1^ copies/µL) of synthetic NSP5 RNA were tested in triplicate. Specificity was evaluated using nucleic acids from non-target enteric pathogens, including norovirus GII, adenovirus, astrovirus, enterovirus, *Escherichia coli*, and *Salmonella* spp., under identical conditions.

### Clinical sample collection and validation

A total of 60 pediatric stool samples were collected from children presenting with symptoms of acute gastroenteritis, including 40 rotavirus-positive cases and 20 rotavirus-negative controls as determined by the reference standard assay. The 20 rotavirus-negative samples included 18 samples from children with diarrhea caused by other enteric pathogens (norovirus, adenovirus, E. coli), confirmed by standard diagnostic methods, and 2 samples from children without gastrointestinal symptoms. The age of the 60 pediatric patients ranged from 6–48 months, with a median age of approximately 18 months. All the samples were collected from the First Affiliated Hospital of Xi’an Jiaotong University. The hospital’s Ethics Committee approved this study (2022–335), and informed consent was obtained from all participants. The nucleic acids were extracted immediately after collection of fresh sample, and the extracts were stored at -80°C until testing. Total viral RNA was extracted using the QIAamp Viral RNA Mini Kit (Qiagen). RT-qPCR targeting the NSP5 gene was used as a gold standard assay. The antigen detection kits were purchased form Sichuan Orienter Bioengineering (20233401736). TtAgo-mediated LAMP results were compared to classic LAMP to assess diagnostic sensitivity, specificity, and accuracy. All data were analyzed with GraphPad Prism 9.0 and MedCalc. ROC curves and AUC values were used to compare assay performance. A *p* value < 0.05 was considered statistically significant.

## Results

### Design and working principle of the TtAgo-mediated LAMP detection system

**Figure 1:**
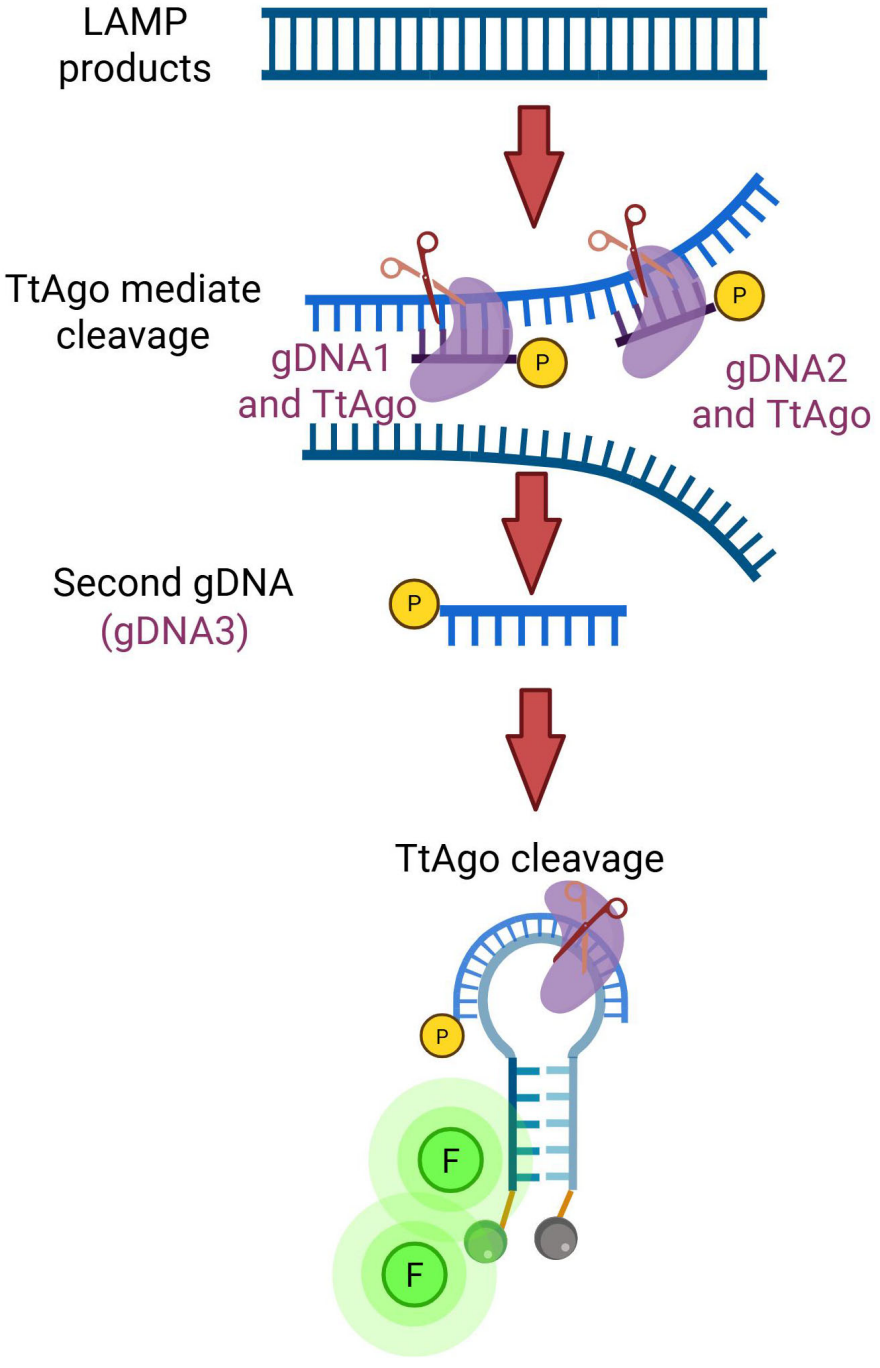
Schematic illustration of the TtAgo-mediated LAMP detection mechanism.

We established a TtAgo-mediated LAMP assay that integrates rapid target amplification with Argonaute-mediated sequence-specific cleavage to achieve sensitive and highly specific detection of rotavirus RNA. As shown in **Figure 1**, reverse transcription LAMP amplifies the conserved NSP5 gene under isothermal conditions, generating abundant double-stranded amplicons.

Phosphorylated DNA guides (gDNA1 and gDNA2) program TtAgo to bind and cleave the LAMP products at defined positions, releasing a short single-stranded DNA fragment. This fragment subsequently acts as a secondary guide (gDNA3), to direct TtAgo to the molecular beacon. Cleavage of the probe disrupts its stem–loop structure and separates the fluorophore from the quencher, resulting in a robust fluorescence signal.

By coupling target-specific amplification with sequential, guide-directed cleavage, the assay effectively suppresses non-specific background signals that commonly occur in classic LAMP, thereby improving diagnostic accuracy. This designed system allows straightforward adaptation to other pathogens by redesigning the primers and guide sequences.

### Optimization of the TtAgo-mediated cleavage reaction

**Figure 2:**
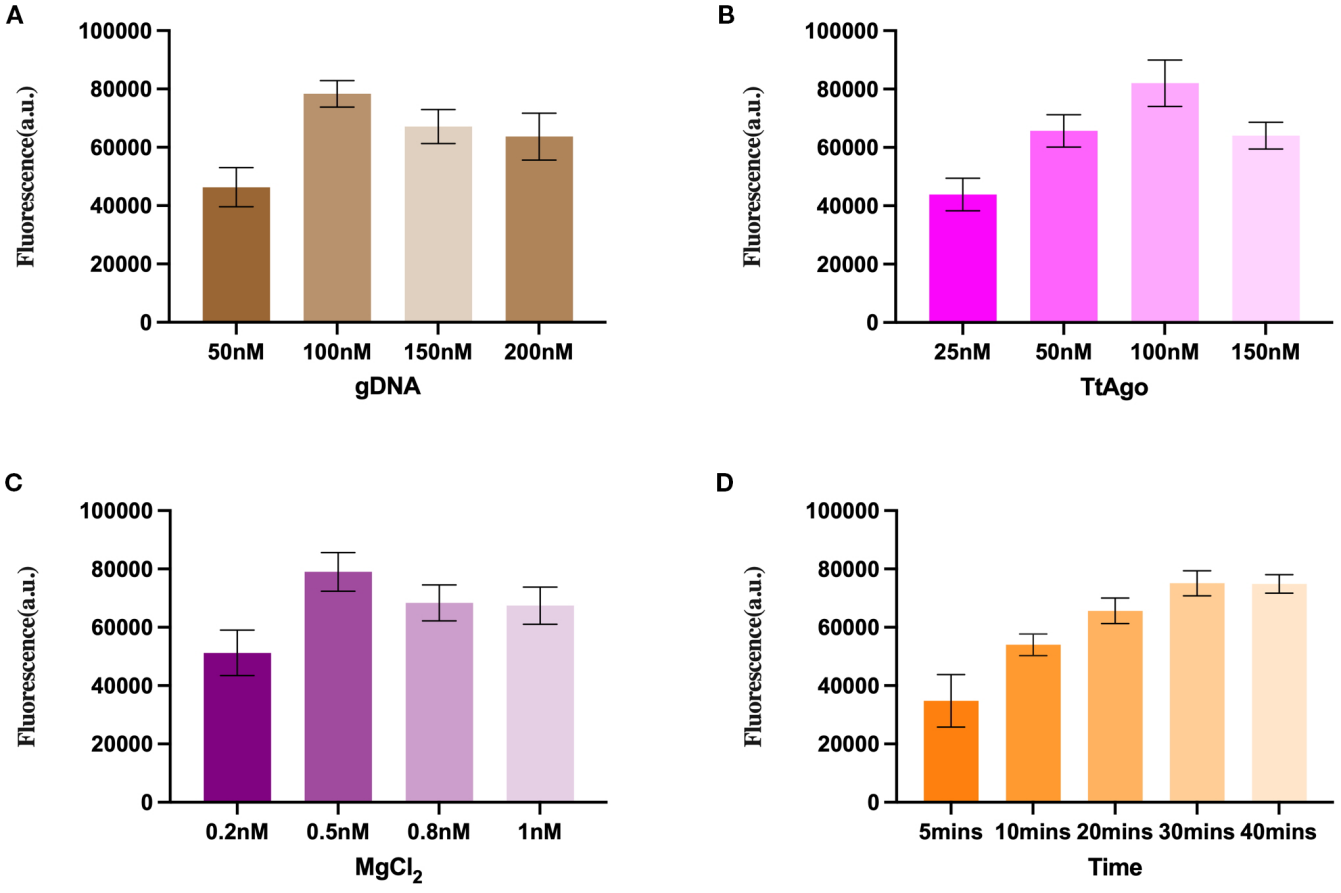
Optimization of the TtAgo- mediated LAMP assay reaction conditions. **(A)** Optimization of gDNA concentration. **(B)** Optimization of TtAgo concentration. **(C)** Optimization of MgCl_2_ concentration. **(D)** Reaction time optimization.

To achieve maximal fluorescence output, we systematically optimized several key parameters of the TtAgo-mediated cleavage system. First, different concentrations of gDNA were tested (**Figure 2A**). Fluorescence intensity increased markedly from 50 nM to 100 nM, reaching its highest value at 100 nM, after which further increases in gDNA concentration did not improve the signal and even led to a slight decline.

Similarly, serial concentration of TtAgo protein revealed that 100 nM produced the strongest fluorescence signal (**Figure 2B**). Lower concentrations resulted in insufficient cleavage activity, while higher concentrations slightly reduced fluorescence, likely due to non-productive complex formation.

Magnesium ions, essential cofactors for TtAgo activity, were evaluated over a range of 0.2–1.0 mM (**Figure 2C**). The optimal signal was observed at 0.5 mM MgCl_2_, whereas both lower and higher concentrations yielded diminished outputs.

Finally, the cleavage reaction time was assessed (**Figure 2D**). Fluorescence intensity increased progressively from 5 min to 30 min, after which the increase plateaued, indicating that 30 min is sufficient to achieve near-complete probe cleavage under the optimized conditions.

These results defined the optimal reaction parameters as 100 nM gDNA, 100 nM TtAgo, 0.5 mM MgCl_2_, and a 30 min cleavage time, which were used for all subsequent experiments.

### Analytical performance of the TtAgo-mediated LAMP assay

**Figure 3:**
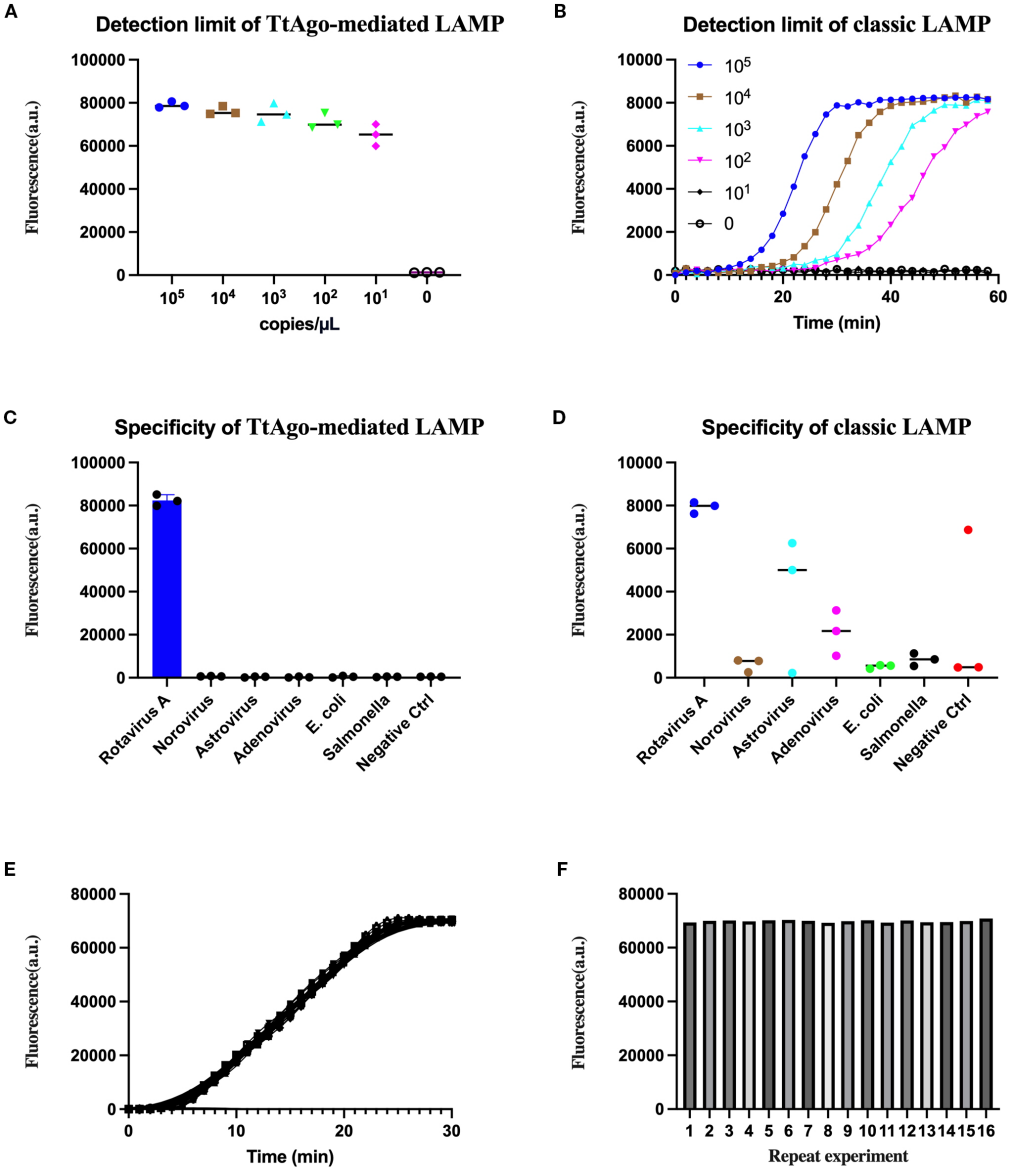
Evaluation of sensitivity, specificity, and reproducibility of the TtAgo-mediated LAMP system. **(A)** Detection limit of TtAgo-mediated LAMP with rotavirus RNA ranging from 10_5_ to 10_0_ copies/μL. **(B)** Detection limit of classic LAMP assessed using the same concentration gradient. **(C)** Specificity of TtAgo-mediated LAMP against rotavirus and other common viral/bacterial pathogens. **(D)** Specificity of classical LAMP against rotavirus and other common viral/bacterial pathogens. **(E)** Real-time fluorescence curves of 16 replicates detecting 10 copies/μL target RNA by TtAgo-mediated LAMP. **(F)** Bar graph showing endpoint fluorescence intensity of each of the 16 replicates.

We first assessed the analytical sensitivity of the TtAgo-mediated LAMP system using serial dilutions of *in vitro*-transcribed NSP5 RNA (10^5^ to 10^0^ copies/μL). A clear fluorescence signal was detected down to 10¹ copies/μL, with no detectable signal in the negative control (**Figure 3A**). In contrast, classic LAMP, monitored in real time, showed reduced sensitivity, with reliable detection achieved only at ≥10^2^ copies/μL and no amplification curves at lower concentrations (**Figure 3B**).

Specificity testing against a panel of common enteric pathogens, including norovirus, astrovirus, adenovirus, *E. coli*, and *Salmonella* spp., confirmed that the TtAgo-mediated LAMP assay produced a strong positive signal exclusively for rotavirus, with all non-targets and the negative control remaining at background levels (**Figure 3C**). Classic LAMP showed occasional low-level fluorescence with non-target templates, indicating reduced specificity and generated false positive (**Figure 3D**).

Reproducibility of the TtAgo-mediated LAMP assay was assessed using 16 independent reactions containing a low template concentration of 10 copies/μL. All replicates generated clear amplification curves within 30 minutes (**Figure 3E**), and endpoint fluorescence intensities remained uniformly high with only minor inter-replicate variation (**Figure 3F**). These findings indicate that the assay maintains strong repeatability and stability even at low target copy numbers.

Taken together, the data indicate that integrating LAMP with TtAgo-mediated probe cleavage not only enhances analytical sensitivity and specificity compared with classic LAMP but also preserves consistent performance across repeated measurements.

### Clinical performance evaluation of TtAgo-mediated LAMP assay

**Figure 4:**
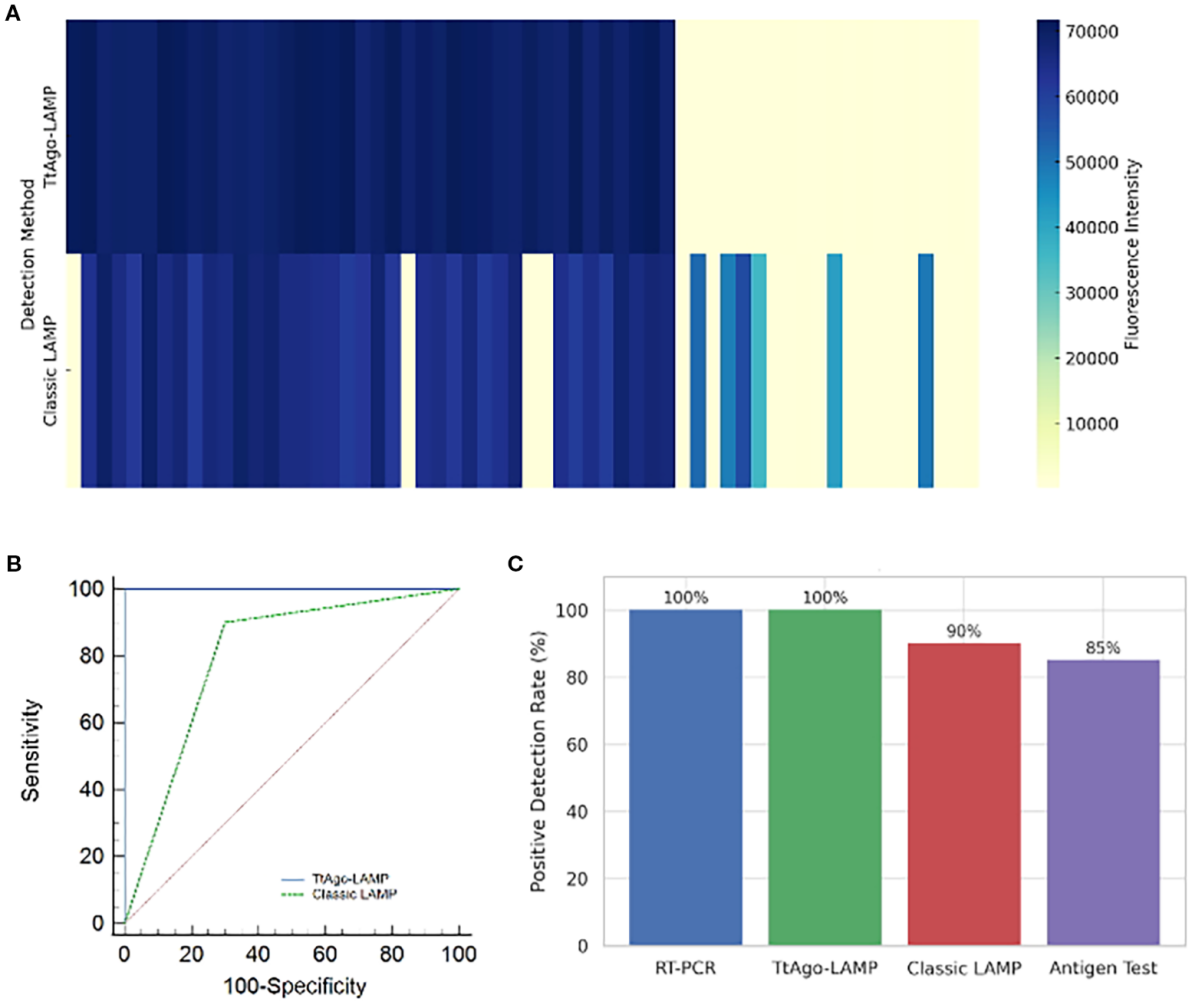
Evaluation of TtAgo-mediated LAMP and Classic LAMP Systems in Clinical Samples. **(A)** Heatmap of fluorescence intensity from clinical samples (n = 60) tested by TtAgo-mediated LAMP and classic LAMP, the horizontal axis indicates different samples. **(B)** ROC curves comparing diagnostic performance. **(C)** Comparison of positive detection rates among four diagnostic methods.

The clinical performance of the TtAgo-mediated LAMP assay was assessed using 60 pediatric stool samples, including 40 rotavirus-positive and 20 negative cases confirmed by RT-qPCR. Fluorescence heatmap analysis showed that the TtAgo-mediated LAMP system yielded uniformly strong signals for all qPCR-positive samples, while all negative samples remained clearly below the detection threshold. In contrast, classic LAMP produced weaker fluorescence in some positives and occasional false results, including both false negatives and false positives (**Figure 4A**). ROC curve analysis demonstrated that TtAgo-mediated LAMP achieved complete separation of positive and negative cases, with an AUC of 1.00, achieving higher performance than classic LAMP, which exhibited a lower AUC due to reduced specificity (**Figure 4B**). When comparing diagnostic effectiveness, both RT-qPCR and TtAgo-mediated LAMP achieved a 100% positive detection rate, whereas classic LAMP and a commercial antigen assay detected 90% and 85% of positive cases, respectively (**Figure 4C**). Overall, these findings indicate that incorporation of TtAgo-mediated LAMP workflow markedly enhances diagnostic accuracy and minimizes the risk of undetected infections.

## Discussion

Earlier studies have highlighted that while LAMP offers rapid nucleic acid amplification under simple conditions, its diagnostic reliability can be compromised by false-positive readouts caused by nonspecific amplification products (Rolando et al., 2020). Several research groups have attempted to address this limitation through CRISPR–Cas–coupled detection, achieving notable improvements in specificity but at the cost of sequence design constraints and the need for RNA guide stability (Li et al., 2019; Lee and Oh, 2022). In contrast, our approach employs TtAgo with a DNA-guided, PAM-independent nuclease, enabling greater flexibility in target site selection and circumventing the preservation issues associated with RNA guides.

Previous applications of Ago-based detection have mainly focused on DNA targets, including bacterial pathogens and DNA viruses (He et al., 2021; Yang et al., 2023). Our results extend this framework to RNA viruses by coupling reverse transcription–based LAMP with TtAgo-mediated molecular beacon cleavage. Compared with earlier rotavirus LAMP assays, which typically report detection thresholds between 10² and 10³ copies/μL, our method achieves an improvement in analytical sensitivity while maintaining high specificity, consistent with or surpassing the gains reported for CRISPR–LAMP systems (Ye et al., 2018b; Gou et al., 2025). This enhanced performance is likely attributable to TtAgo’s rigorous sequence-specific recognition criteria, which enable selective elimination of off-target amplicons before signal generation. Given its reliance on sequence-specific guide DNAs and isothermal amplification, the TtAgo-LAMP platform could be readily adapted for the detection of other clinically relevant RNA viruses, such as norovirus or dengue virus, by redesigning primers and guide sequences to target conserved genomic regions.

From a real clinical setting, reducing false-positive results is particularly important in pediatric gastroenteritis diagnosis, where unnecessary interventions can impose avoidable clinical and economic burdens (Tilmanne et al., 2019). In our evaluation, the TtAgo-mediated LAMP assay demonstrated complete concordance with RT-qPCR across a mixed cohort of positive and negative stool samples, outperforming both classic LAMP and commercial antigen tests. The ability to maintain sensitivity at low viral loads also positions this method as a valuable option for early detection, an aspect critical for limiting rotavirus transmission under epidemic conditions.

Despite its merits, the assay still leaves room for refinement. The current two-step setup, although beneficial for maintaining enzyme efficiency and reducing nonspecific reactions, is less convenient than fully consolidated single-tube formats. Refining buffer and reagent conditions to support integrated operation would streamline the workflow. A more extensive assessment across diverse rotavirus genotypes and field-derived samples will be valuable for verifying assay robustness, whereas considerations of cost, feasibility, and scalability in resource-limited settings will be pivotal for its routine implementation.

In conclusion, this study established a TtAgo-mediated LAMP assay as a robust, high-specificity alternative to classic LAMP and a practical complement to CRISPR-based detection systems. By integrating programmable DNA-guided cleavage with isothermal amplification, the assay effectively addresses one of the major limitations of LAMP while preserving its rapid turnaround time. With further optimization toward full integration and broader validation, this platform could be extended to other clinically relevant RNA viruses, enhancing the reach of molecular diagnostics in diverse healthcare settings.

## Data Availability

The original contributions presented in the study are included in the article/supplementary material, Further inquiries can be directed to the corresponding author/s.
